# Does a Traceability System Help to Regulate Pig Farm Households’ Veterinary Drug Use Behavior? Evidence from Pig Farms in China

**DOI:** 10.3390/ijerph191911879

**Published:** 2022-09-20

**Authors:** Zengjin Liu, Ning Geng, Zhuo Yu

**Affiliations:** 1 Shanghai Academy of Agricultural Sciences, Shanghai 201403, China; 2 School of Public Administration, Shandong Normal University, Jinan 250014, China; 3 School of Management, Ocean University of China, Qingdao 266100, China

**Keywords:** pork traceability system, safety effect, pig farm households, safety behavior

## Abstract

In China, there is a renewed interest in traceability systems as an efficient tool to guarantee pork safety. One of the pathways through which a traceability system can benefit consumers is by easing information asymmetry. However, past literature on the traceability system in China pays more attention to theoretical analysis and less to empirical analysis. Using a large-scale survey of pig farms in China, we investigate the effects influencing farmers’ participation in the traceability system. Findings show that a traceability system can influence the safety of pork indirectly through its impacts on farmers’ production behaviors. Another important finding is that unsafe pork is a result of non-standard use of veterinary drugs, and the traceability system works well for farmers by pushing them to take stricter safety measurements.

## 1. Introduction

Food safety is a worldwide public health issue [1], which often results from information asymmetry between producers and consumers [2]. Specific market and production characteristics of food supply chains are motives for vertical coordination in order to gain competitive advantage. Quality assurance systems will contribute as a facilitating factor [3,4]. In general, the implementation of food traceability should be associated with effective ways of communicating traceability information to the consumers and other stakeholders [5]. With the rapid development of information technology, building a “from farm to fork” food traceability is considered as the best way to resolve information asymmetry, to eliminate consumer’s trust crisis, and to ensure food safety [6]. Pork is one of the major meat products consumed in China. Recently, many events related to pork and public health, such as African swine fever (ASF), swine flu, and the Clenbuterol Event (improper use of lean meat powder), have had a serious impact on pork supply and consumption. Thus, the safety of pork is of crucial strategic significance to China’s food safety. The safety of pork is a concern to several public authorities and value chain actors [7,8,9]. Although the pork safety problem is widespread in the world, due to the long industrial chain of China’s pork industry, the large number of small-scale farmers and retail investors, and the complex interest relationship, it is more likely to produce some pork safety problems. The Chinese government is striving to execute stringent and effective food regulations to meet the demand of food safety and to prevent frequent food safety scandals in the pork industry. A food traceability system is a powerful solution to food safety problems [10,11] and has become the main tool and premise to ensure meat quality and to prevent food safety risks.

Traceability is one of the test/measurement indicators in food regulations, and the Chinese government has put tremendous human and financial resources into constructing a food traceability system. At present, it has become a prerequisite for ensuring meat safety and reducing consumer trust crisis. Traceability is applied as a tool to assist in the assurance of food safety and quality as well as to achieve consumer confidence [12,13]. Traceability information covers the whole food chain, including livestock farming, slaughtering, processing, distribution, and marketing. Due to the efforts of the Chinese government in the construction of the traceability system, Chinese consumers’ willingness to pay (WTP) for traceability information and quality certification significantly improved [14,15,16].

Improper use of veterinary drugs by pig farmers may affect food safety and the agro-ecological environment [17]. Given the frequent food safety scandals of the pork industry, we found many of China’s food safety issues can be tracked back to the upstream farmers, as some farmers still depended heavily on the abuse of chemical substances, including additives, antibiotics, and hormones (e.g., clenbuterol or “lean meat” hormone) to cope with various breeding problems in the meat industry [18]. As we know, conventional poultry farms use antibiotics extensively, which contributes to the rise of antibiotic-resistant pathogens [19]. Meanwhile other health threats, such as antibiotic-resistant strains of staphylococcus aureus, have emerged from pig farms as well. In an effort to keep the food supply safe, integrated and intensive efforts to improve food safety among pork value chain actors should be taken [20,21]. China’s government has executed a series of tougher food safety laws [22] to control pig farmers’ production behavior. Food traceability systems have been widely carried out in some developed and developing countries and have an essential function in food safety guarantee. FTS (short for Food Traceability System, are defined consistently elsewhere in the paper) has become increasingly important and relevant over time in its role to maintain and ensure the safety and integrity of the Chinese food supply [23].

The promotion of safe pork production is extremely important and has attracted the attention of academics. Ji et al. have found that cooperative membership has a significant and positive influence on farmers’ propensity to adopt safe production practices [24]. Meanwhile, some academics have studied consumers’ willingness to pay for pork traceability information [25,26]. However less is known about whether the traceability system works for pig farmers, especially the rational use of food additives, and how the traceability system influences the behavior of pig farmers. Although China’s FTS has made some progress, including policy support, project implementation, there are still many challenges that need to be overcome [27,28,29]. The real problem is that the length of the pig industry chain and the complicated interest relationships make an obstacle in the progress of implementing the pork traceability system [30,31]. After combining relevant literature, it was found that existing studies did not address the issue of “What is the actual effect of the safety effect of a pork traceability system?” or “Does the pork traceability system help to standardize the safety behavior of pig farmers?” This is a major issue with practical significance and needs to be empirically explored and verified. Hirschauer et al. [32] incorporated social norms and community pressure into the analysis of a variety of behavioral factors affecting producers’ motivation to deliberately violate food safety norms. Ji’s research constructed a government contract incentive model and a market reputation mechanism model for theoretical discussion, and conducted an empirical verification via using two typical cases of pig slaughtering and processing enterprises in a Beijing field investigation in an attempt to explore the role of the pork traceability system in ensuring pork safety [33]. The results showed that the pork traceability system regulates the safety behavior of slaughter companies via the enhancement of safety monitoring and reputation mechanism; the government supervision and improvement of supervision efficiency brought by the construction of a pork traceability system can help to curb moral hazard activities and the opportunistic behavior of slaughter enterprises. The reputation mechanism can solve pork safety problems as well as the explicit incentive mechanism to motivate and restrict slaughter enterprises’ safety behavior, but it is also affected by the level of pork traceability. An increasing number of countries have started animal product traceability system projects, traceable animal products are gaining more market share in the global supply chain, which can provide consumers with quality assurance throughout the supply chain [34]. Access to industry chain information through QR code scanning can enhance consumers’ knowledge of pork product quality and safety and promote stable social development [35,36]. The pork traceability platform in Tianjin, China, can integrate online all pork identification and related quality data from farming and slaughtering to marketing, and achieve all-round traceability of pork from origin to consumption process. The implementation of the pork traceability platform in Tianjin will provide technical support to ensure the quality and safety of pork production and meet consumer demand [37]. Therefore, the above-mentioned study has provided a solid theoretical basis for the study of this paper. However, that study only used two pig slaughtering and processing companies as examples to empirically analyze the actual effect of the safety impact of the pork traceability system, without regulating the safety behavior of other stakeholders in the pig industry chain.

Since the mid-to-late 1990s, especially with the Olympic Games and the World Expo as a starting point, China has begun to explore the construction of a food traceability system that has achieved remarkable achievements. The new Food Safety Law, which came into effect on 1 October 2015, made clearer provisions for establishing a food safety traceability system. The traceability system of agricultural products by the Ministry of Agriculture and the Meat and Vegetable Distribution Traceability System of the Ministry of Commerce have greatly promoted the construction of the pork traceability system. The main goal of the current pork traceability system construction is to trace the source, and related work is also carried out around this goal. Therefore, the role of pork traceability system construction is mainly to bring conceptual changes and participation to the stakeholders in each link of the pig industry chain. The traceability system can increase stakeholders’ trust in the ability to trace the origin of pigs and pork, make them aware of the risks and costs of violating the laws and regulations, and help to regulate their safety behavior, thereby supporting the improvement of pork safety. It should be recognized that the key to the above-mentioned role lies in whether the stakeholders in each link of the industry chain know or recognize that their pig farms, slaughter plants, or sales stalls participate in the traceability system, which in turn depends directly on the construction work that government dedicates to the pork traceability system. In addition, traceability in agri-food has become a focus of study among related scientists and an important aspect of consumer awareness that demand foods that are available be of good quality, healthy, and traceable in handling processes [38].

Studies have shown that the pig industry chain includes pig breeding, distribution, slaughtering and processing, pork sales, and other links. The behavior of stakeholders in all links of the industry chain will affect pork safety [31,32]. For example, the service quality of cooperatives has a positive impact on farmers’ production safety behavior [33]. As the source of the pork supply chain, pig farm households played a substantial role in ensuring pork safety [38]. In combing the relevant research literature on farmers and traceability systems, we found that more scholars paid attention to the research field of farmers’ participation in traceability system. There is relatively little literature on the role of traceability systems for farmers. This paper mainly discusses the effect of traceability system on farmers in other industries (compared with pork industry) from the theoretical or empirical level: first, it further optimizes the quality of product production through data accumulation or sharing; second, it improves the income of farmers; and third, it improves the technical efficiency of production [39,40,41]. Therefore, this paper attached more attention to the safety behavior of pig farm households and the impact of the construction of a pork traceability system on their safety behavior. If the construction of a pork traceability system has a positive effect on the safety behavior of pig farm households, then it can be considered that the pork traceability system helps to ensure pork safety from the source. Based on this, this paper used questionnaire data from a survey of 396 pig farm households in Beijing, Henan and Hunan provinces to empirically analyze the safety behavior of pig farm households and their influencing factors. To know the mechanism and effect on the safety behavior of pig farm households, with a view to answering and empirically verifying “Does the pork traceability system help to ensure pork safety?” This is a question of great practical significance and countermeasures are proposed, which will help to provide an objective basis for promoting the construction of a pork traceability system and ensuring pork safety. According to the study purpose and ideas, a questionnaire was designed that mainly included the following parts: the first part was the basic characteristics and basic business conditions of the pig farm households; the second part was the vertical coordination situation of the pig farms’ production and management; the third part was the safety control situation of the pig farm households; the fourth part was the safety cognition and supervision of the pig farm households; and the fifth part was the cognition and behavior of the pig farm households participating in the pork traceability system. Then, in the second part of the paper, we conducted the theoretical analysis of the research problem and the construction of the empirical model. In the third part of the paper, the questionnaire survey and data are briefly analyzed. In the fourth part of the paper, the econometric model is used to further verify the theoretical hypothesis, and the fifth part of the paper, combined with the model results, summed up the research conclusion.

## 2. Methodology

### 2.1. Theoretical Analysis

There are many studies on the influencing factors of the safety behavior of pig farm households. Scholars generally believe that the factors that affect the safety behavior of pig farm households include the basic characteristics of individual farm households, basic breeding conditions, vertical coordination models, safety cognition, and external supervision [42,43,44,45]. Studies have been conducted to illustrate that poultry traceability systems are being expanded in the areas of production, processing, and distribution to meet consumer concerns about poultry public health and other issues [46]. However, existing studies have not analyzed the influence of a pork traceability system on the safety behavior of pig farm households [42,43,44,45]. Studies have also shown the impact of government’s food safety inspection on product quality improvement, traceability related cognition, consumer preference, education level, and other influences on the adoption of a traceability system [47,48,49,50].

In view of this, in addition to examining the influence of the above factors on pig farm households’ safety behaviors, this paper also tried to clarify the mechanism of the pork traceability system on it and to quantitatively analyze the extent of the effects.

The ultimate purpose of the pork traceability system is to ensure pork safety. In this sense, it can be defined as a safety strategy, but the immediate goal of the current pork traceability system is to realize traceability. How traceability ensures pork safety is the key issue that needs to be clarified. “Traceability” is the core concept of food traceability systems [45,47]; the European Union defines it as the availability to trace and to follow a food, feed, animal, or substance through all links of production, processing, and distribution. The definition of “traceability” essentially reflects the traceability of food. In terms of pork in China, its traceability can be divided into four levels, which are traced back to pork sellers, pig slaughtering and processing enterprises, pig farm households, pig breeding feed, and veterinary drug use, by which the difficulty is gradually increased accordingly. The most direct meaning of traceability to consumers is to protect their rights and interests. For instance, the right to know is one of them, which is helpful to reduce information asymmetry and to solve market failure. The most direct significance for producers is to clarify responsibility, so that producers have a clearer understanding of the consequences of their actions before engaging in illegal acts.

**Figure 1:**
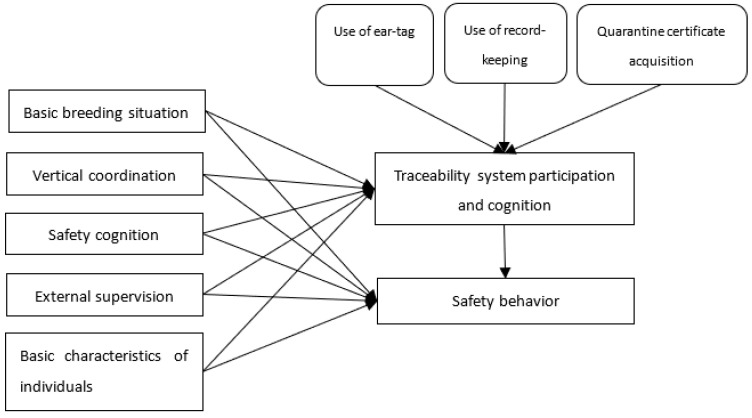
Theoretical model framework.

The drivers differ across FTSs depending on the specific information needs of stakeholders along the supply chain [51]. The establishment of a pork traceability system can significantly improve the ability of pig purchasers and sellers, pig slaughtering enterprises, pork sellers, and pork consumers to trace back to pig farm households, which helps to strengthen the regulatory and reputation incentives brought to regulate the safety behavior of pig farm households [35]. In reality, the key to the role of safety assurance is to make pig farm households recognize that their pig farms are involved in the pork traceability system, and this directly depends on government efforts, such as use of ear-tags, use of record- keeping, and quarantine certificate acquisition [34,52]. The above two aspects will increase pig farm households’ trust in traceability capabilities, make them aware of the risks and costs of illegal activities, and thus regulate their safety behavior. Only when the farm households believe that their pig farms have participated in the pork traceability system can the enhanced regulatory and reputation incentives brought by the traceability system work to regulate their safety behavior [53]. Therefore, this study proposed the following research hypotheses: the safety behavior of pig farm households who believe that their farms have participated in the pork traceability system is significantly higher than those who do not. The pig farm households that implement use of ear-tags, use of record-keeping, and quarantine certification acquisition are quite sure that their pig farms have participated in the pork traceability system. Based on this, the theoretical model framework of Figure 1 is formed.

### 2.2. Empircal Model

Assume that the safety behavior of the pig farm households (specifically refers to the use of veterinary drugs) is determined by a potential utility level variable U. When the utility level is lower than $U^{*}$, the farmer will choose to regulate the use of veterinary drugs, and the farmer will not choose to regulate the use of veterinary drugs if the utility level is higher than $U^{*}$. The use of veterinary drugs by farm households can be represented by the following probability model:

$$\mathrm{Pr}ob(Y=1)=\mathrm{Pr}ob(U>U^{*})$$

$$\mathrm{Pr}ob(Y=0)=\mathrm{Pr}ob(U\leq U^{*})$$

Among them, the potential utility level variable is jointly determined by factors, such as participation cognition of traceability system, basic breeding situation, vertical coordination, safety cognition, external supervision, and individual basic characteristics, that is $U=\beta_{0}+XB+\mu$, X affects the utility of the farm households and their use of veterinary drugs. The probability function of this model is calculated based on a standard normal distribution, that is $\mathrm{Pr}obit(Y)=\phi (\beta_{0}+XB)$. Hence, the model that needs to be estimated can be transformed into the following binary probit model:

(1) $$Y=f_{1}(T,G,Z,C,J,P,\mu_{1})$$

Among them, the explained variable Y represents the behavior of using veterinary drugs in pig farms. 1 means that veterinary drugs are not used in a proper way, that is, banned drugs are used. The variable T represents the judgment and perception of pig farmers on whether they participate in the traceability system, and its value is 0 or 1. Pig farm households who believe that their pig farms have participated in the pork traceability system are represented by 1, and the others are represented by 0. Among the other explanatory variables, G is basic breeding variable, including working experience, breeding scale, breeding mode, and slaughtering number; Z is vertical coordination model variable, including farm households professional cooperatives, pig sales mode, pig sales relationship, feed procurement mode, feed procurement relationship, veterinary drug procurement mode, and veterinary drug procurement relationship; C is safety cognition variable, including the understanding level of regulations on feed additives and using veterinary drug; J is external regulatory variable, including detection level cognition, purchasers’ supervision and government supervision; P is individual basic characteristics variable, including gender, age, and education background; $\mu_{1}$ is residual term.

In addition, according to the previous theoretical analysis, a conclusion can be summarized that the participation of pig farm households in a pork traceability system is affected by government implementation in use of ear-tags, use of record-keeping, and quarantine certificate acquisition. Based on this, the following model is established:

(2) $$T=f_{2}(IV,G,Z,C,J,P,\mu_{2})$$

Among them, IV including use of ear-tags use of record-keeping and quarantine certificate acquisition; $\mu_{2}$ is residual item.

The independent variables of the model are defined in Table 1.

## 3. Survey and Data

**Figure 2:**
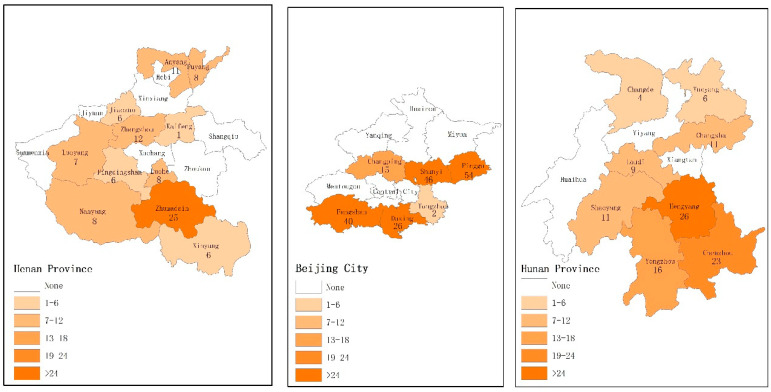
Geospatial distribution of the sample. Note: the central urban area of Beijing City includes six urban areas, namely Haidian, Chaoyang, Dongcheng, Xicheng, Shijingshan, and Fengtai.

The data of this study was based on questionnaire survey data of pig farm households across Beijing, Henan, and Hunan provinces. Finally, 410 questionnaires and 396 valid questionnaires were obtained of which 183 were from Beijing, 98 were from Henan, and 115 were from Hunan. This research and investigation was mainly divided into two stages, both of which were face-to-face surveys. Firstly, based on the platform of Beijing innovation group of the pig industry technology system, from March to August 2014, work was made in conducting the survey in six suburbs of Beijing, including Daxing, Pinggu, Fangshan, Shunyi, Tongzhou, and Changping, and the interviewed pig farm households included the vast majority of all pig farm households in Beijing. Secondly, the fixed cooperative observation point platform of the Rural Economic Research Center of the Ministry of Agriculture and Rural Affairs was used to conduct the questionnaire survey in December 2017 in 11 prefecture-level cities, including Kaifeng, Hengyang, Chenzhou, Yongzhou, Shaoyang, Changsha, Loudi, Zhuzhou, Yueyang, Changde, Huaihua, and Xiangtan across Hunan Province, and the interviewed pig farm households were all fixed survey objects every year. See Table 2 for sample distribution and Figure 2 for geospatial distribution of the sample.

Basic characteristics of the samples are shown in Table 3. From the perspective of gender, the majority of the respondents are male, accounting for 81.31%. From the perspective of age, the respondents aged 40–59 accounted for 77.78% of the total sample number, people aged 18–39 only account for 10.10%, and aged 60 years old and above account for 12.12%. Pig breeding is a heavy task that the young are reluctant to engage in, and it is also difficult for the old, so most practitioners are middle-aged people. From the perspective of educational background, nearly half of the respondents have a junior high school education or below, 43.69% of them have a high school/technical secondary education, and 11.87% of them have an/a undergraduate/junior college education. In general, the education level of pig farm households is low, this is also a common status quo in the pig industry. In terms of working experience, 23.74% of the respondents have been engaged in pig breeding within 10 years, 59.34% of them have been employed for 10–19 years, and only 16.92% of the respondents have been employed for more than 20 years. Pig breeding is a job that needs a long period of profitability and requires experience accumulation. Despite that, pig breeding work is tiring, the fixed cost investment is large, and the opportunity cost of the practitioner is not high for which most of the practitioners have spent a long time in pig breeding. From the perspective of breeding scale, the number of female pigs in 12.88% of the farms is below 10, the number of female pigs in 36.36% of the pig farms is between 10–49, the number of female pigs in 20.20% of the pig farms is 50–99, and the number of female pigs in 30.56% of the pig farms is up to 100 and above. From the perspective of breeding mode, 43.69% of the pig farms adopted all-in and all-out farming mode. Judging from the number of pigs sold, 46.97% of the interviewed pig farms had less than 50 pigs per each time on average. If the number of pigs sold is too low, it will bring great difficulties to the construction of the pig traceability system, and there is a link between breeding scale and breeding mode.

## 4. Model Estimation Results and Analysis

### 4.1. Descriptive Analysis of the Veterinary Drug Use Behavior and Participation of Traceability System in Pig Farms

Generally, it is believed that the hidden dangers of pork safety mostly arise from the pig breeding sector. The main stakeholders in the pig breeding sector are pig farm households. The hidden dangers of pork safety during this stage are mainly sales of pork from diseased pigs, water-injected pork, and use of banned drugs and high drug residues. According to the results of our investigation and analysis, it is known that the hidden dangers of pork safety are mainly due to the improper use of veterinary drugs in pig farms. Therefore, this paper regarded the use of veterinary drugs by pig farm households as a specific measure to study their safety behavior.

In order to maintain the health of the pigs and to promote growth of the slaughtering number, the dilemma of using veterinary drugs is faced by almost all pig farm households, and the key point is whether they are used within standards. Whether to use veterinary drugs is regulated mainly from pork safety. Before further analysis of the use of veterinary drugs in pig farms, clarifications are necessary, including their three main uses, which are: first, for preventing disease; second, for treating diseases; and third, for feed additives. Feed additives can be divided into nutritional additives and non-nutritive additives, the latter including growth promoters, insect repellent health care agents, drug preservation agents and other additives among which growth promoters are the fundamental drug. On this basis, we will talk about the norms for veterinary drug use. The irregular use of veterinary drugs includes three categories: first, the use of banned drugs; second, not carried out drug withdrawal times. Among them, the use of banned drugs is the most harmful behavior to consumers. The survey found that 31.57% of the interviewed pig farm households had used banned drugs in the past years. The question was designed as “Which of the following feed additives or veterinary drugs have been used on pig farms since last year? (Multiple choices)”, the options included: (1) microecologics, (2) clenbuterol, (3) ractopamine, (4) vitamins, (5) salbutamol, (6) oxytetracycline, (7) chloramphenicol, (8) diazepam, (9) sulfadiazine, (10) chlorpromazine, (11) methyltestosterone, (12) iodide casein, (13) norfloxacin, (14) estradiol benzoate, (15) zeranol, (16) streptomycin, (17) cephalexin, (18) antibiotic residue, (19) diethylstilbestrol, (20) furazolidone, (21) carbofuran, (22) lindane, (23) nandrolon phenylpropionate, (24) metronidazole, (25) dimetridazole, (26) sodium nitrophenolate, (27) used other drugs (Please fill in the drug name). It should be recognized that the use of banned drugs is a very sensitive issue. If respondents are directly asked if they have used banned drugs, then a large number of the respondents who have used banned drugs will not admit the use. Perceived risks and cautious behavior regarding antibiotics were important predictors of farmers’ perception of policy measures to reduce antibiotic usage [54]. Meanwhile, some respondents may actually use the banned drugs without knowing it is banned. Based on this, this study adopted the following measures to reflect as closely as possible the use of banned drugs by pig farm households. On the one hand, the questionnaire was designed not to directly ask farm households whether they have used banned drugs. In accordance with the announcement of the Ministry of Agriculture (No.176 and No.193) on banned drugs and by adding some nutritional feed additives and allowed veterinary drugs as options, respondents were able to choose from them. The banned drugs listed in the questionnaire included the main types of banned drugs in relevant regulations of the Ministry of Agriculture, especially stimulants, such as clenbuterol, ractopamine, and salbutamol; on the other hand, during the questionnaire survey, it was explained to the respondents that the survey results were only used for research projects, not for other purposes, to dispel the doubts of the interviewees in order to obtain the most authentic and reliable results possible. Meanwhile, it should also be noted that in order to ensure the safety of animal products and to maintain public health, the Ministry of Agriculture has continuously increased the risk assessment and safety re-evaluation of veterinary drugs in recent years. In the past three years, eight veterinary drugs have been banned from being used in food. For animals, especially four species of humans and animals, lomefloxacin, pefloxacin, ofloxacin, and norfloxacin were banned from being used in food animals in 2015. Due to this questionnaire survey involving two stages, “norfloxacin” in the questionnaire title option was not a banned drug in the survey year 2014, but it was already a banned drug in the survey year 2017. This study defined whether the use of banned drugs strictly follows the relevant regulations in reality.

### 4.2. Participants’ Cognition and Behavior on Pork Traceability System

The survey found that 295 pig farm households interviewed stated that they knew “traceable pig and pork traceability system” or “traceable pork” before the survey, accounting for 74.49% of the total sample of which 163 people believed that their pork had participated in the pork traceability system, which accounted for 41.16% of the total sample number. It can be seen that the cognition of the pig traceability system is relatively high, which is closely related to the vigorous promotion of the pork traceability system. In addition, use of ear tags, use of record-keeping of breeding, and quarantine certification are the basic tasks related to whether the construction of a pork traceability system can be successfully advanced [55], among which the pig ear tags and pig quarantine certification are the most direct and valid credentials of traceable sources. The survey found that 72.98% of the pig farm households surveyed said that all fattening pigs raised on their farm were equipped with ear tags but 27.02% of the fattening pigs of the pig farms had more or less no ear tags or ear tags falling off. A total of 91.16% of the pig farm households surveyed stated that pig farms have pig breeding archives or epidemic prevention archives; 81.57% of the pig farm households surveyed said that they obtained animal quarantine certificates when they sold pigs. What needs to be explained here is that the purchase of pigs by pig purchasers and sellers is based on the number of trips (batch). Generally speaking, a large transport truck can load 100–200 pigs, and a small transport truck can load 50–100 pigs. At present, the number of pigs produced by each pig farm household is not same. Therefore, the pigs of each trip may belong to several pig farm house-holds. The pig buyers and sellers will issue the full number of pigs and then be issued animal quarantine certificate by the supervision department, which caused the pig farm households to say that they have not obtained a pig quarantine certificate. Although the pigs have also been quarantined in this case, due to the differences in the safety behavior of different farm households, and the quarantine is conducted on a batch basis. This situation not only brings obstacles to the construction of a pig and pork traceability system but also results in some hidden dangers of pork safety. Regarding certain issues, especially in terms of wearing ear tags and obtaining quarantine certificates, we need to strengthen and strictly implement supervision.

### 4.3. Quantitative Analysis of Factors That Affect the Safety Behavior of Pork Sellers

The foregoing Equations (1) and (2) constitute a simultaneous equations system. If there is a correlation between the residual terms of the two equations above, the single equation estimation mode is not the most efficient. There is no correlation between the residual terms, so it is feasible to estimate Equations (1) and (2) separately [56]. In view of this, this paper first performed a Hausman test on the correlation between the residual terms of Formula (1) and Formula (2). The test results showed that the likelihood ratio of Rho = 0 is 13.975, and the corresponding *p* value is 0.0002. The null hypothesis is rejected under the 1% significance level, which indicated that the variable has strong endogeneity. This paper used stata13.0 to select the finite information maximum likelihood mode (LIML) to estimate the bivariate Probit model composed of Equations (1) and (2) [57]. Meanwhile, in order to enhance the reliability and persuasiveness of the model estimation results, the binary Probit model is estimated on the factors affecting the safety behavior of pork sellers, without considering the endogeneity problem of traceability variables. The results are shown in Table 4. Average marginal effects of the variables in the model are shown in Table 5.

#### 4.3.1. The Results of Empirical Verification on Whether the Pork Traceability System Helps to Guarantee Pork Safety

It can be known from the model estimation results that the participation of the traceability system significantly affects the veterinary drug use behavior of pig farm households, and use of ear-tags significantly affects their participation cognition of the traceability system. That is, if their own farms have participated in pork traceability, the probability of non-standardized use of veterinary drugs (use of banned drugs) is lower than that of pig farm households who believe that their pig farms are not involved in the pork traceability system; using ear-tags directly affects whether pig farm households believe that their pig farms participate in the pork traceability system. Compared to those whose pig farms that do not equip the fattening pigs with ear tags raised on their farms, those whose pig farms equip the fattening pigs with ear tags are prone to believe that their own farms have participated in the pork traceability system. This is a solid test of the research hypothesis in this paper and confirms that the safety behavior of pig farm households who believe that their farms have participated in the pork traceability system is significantly higher than that of farms that do not participate in the pork traceability system. Using ear-tags indirectly affects their safety behavior by directly influencing pig farm households’ participation cognition. Besides, the above results verified that the construction of a pork traceability system indeed helps to guarantee pork safety from the source. This is in line with the findings of Adam’s research into traceability systems for safe beef production and product quality [58].

#### 4.3.2. Influence of Other Variables on Veterinary Drug Use Behavior of Pig Farm Households

In addition to the cognition variables involved in the traceability system, the three variables, such as farming mode, pig sales mode, and veterinary drug purchase mode, also significantly affect the veterinary drug use behavior of pig farm households.

First, the rearing mode significantly affects the use of veterinary drugs by pig farm households. That is, the use of veterinary drugs by them that adopt the all-in and all-out mode is more standard than that of pig farm households that do not. Compared with pig farms adopting non-all-in and non-all-out, the management condition of pig farms adopting all-in and all-out farming mode is relatively high, environmental sanitation conditions are cleaner and tidier, pig disease incidence is lower, and disease control is more accurate and timelier, so its veterinary drug use behavior is relatively more standardized. This is in line with the findings of Pedersen’s research that all-in and all-out mode has a positive influence on both health and growth [59].

Second, pig sales mode significantly affects the veterinary drug use behavior of pig farm households. That is, compared to the veterinary drug use behavior of pig farm households who sell pigs mainly via agreements or integrated mode, the probability of regulation is higher in that of pig farms that sell pigs via market free trading. This is inconsistent with the expected direction of action. The possible reason is that on-site grading settlement is between the pig dealer and the pig farm households. The post-mortem grading settlement that includes carcass rate, lean and fat, water content, etc. is between the pig slaughtering enterprise and the pig dealers. Therefore, in the context of free market transactions, pig purchasers and sellers have sufficient motivation to strictly control the safety behavior of pig farm households. However, in the case of tight pig sources, pig purchasers and sellers will reach a pig procurement agreement (in oral or written form) with pig farm households in order to ensure pig sources. There are many small-scale pig farms or retail investors. The requirements of pig farm households for safety behavior will also be reduced, thereby increasing the probability of irregular use of veterinary drugs.

Third, the veterinary drug procurement mode significantly affects the use of veterinary drugs by pig farm households. That is, compared to the use of veterinary drugs by pig farm households who purchase veterinary drugs mainly via free market transactions, the probability of being standard is higher in pig farms that purchase veterinary drugs via agreements or integration. Households are less likely to be more regulated. China has established a stricter veterinary drug sales management system, and sellers who sell veterinary drugs through agreement or integration with pig farm households are more formal. They are usually registered with relevant government departments and strictly regulated, and they are sold through free market transactions. Veterinary drug sellers are more likely to have small-scale, poorly managed veterinary drug stores, and the government has weak supervision over them, which is more likely to result in the sale of banned drugs. This is in line with the findings of Ning’s research [60].

#### 4.3.3. Analysis of the Influence of Other Variables on Traceability System Participation Cognition of Pig Farm Households

In addition to the use of ear-tags variable, five variables including breeding scale, professional cooperatives, government supervision, gender, and age significantly affect the cognition of pig farm households’ traceability system participation.

First, breeding scale significantly affects the cognition of pig farm households’ traceability system participation. That is, compared to pig farm households with less than 50 female pigs that can breed at the end of the year, those whose farms had more than 50 female pigs that could breed believed that it was more probable that their pig farm had participated in the pork traceability system. Second, professional cooperatives significantly affects the cognition of pig farm households’ traceability system participation. That is, compared to pig farm households who have not joined professional cooperatives, those who join a professional cooperative have an even smaller probability of believing that their pig farms have participated in the pork traceability system. Third, government supervision is significantly affecting the perception and participation of pig farm households in the traceability system. That is, pig farm households that believe the government has strong detection and punishment in pork safety are considered to be more likely to participate in the pork traceability system. This is in line with the findings of Jin’s research that government regulation helps pig farmers to participate in the traceability system [61]. Finally, gender significantly affects pig farm households’ participation in the traceability system. That is, male respondents are more prone to believe that their pig farms participate in the pork traceability system; age inversely significantly affects pig farm households. The cognition of the traceability system is that older respondents are less likely to believe that their farms participate in the pork traceability system.

## 5. Conclusions

This paper mainly raised the question of whether the construction of a pork traceability system could help to improve pork safety and questionnaire data from 396 pig farm households in Beijing, Henan, and Hunan was used to verify this question. Studies have confirmed that the pork traceability system helps to improve pork safety. Using ear-tags indirectly affects the safety behavior of pig farm households by directly affecting their cognition of the traceability system. That is, the pig farm households who equip fattening pigs with ear tags are more prone to believing that their pig farms have participated in the pork traceability system than those who do not equip fattening pigs with ear tags, and the probability of non-standardized use of veterinary drugs by pig farm households who believe that their pig farm has participated in the pork traceability system is lower than that of pig farm households who believe that their pig farm has not participated in the pork traceability system.

This paper also draws other conclusions: there is a hidden danger to safety in the pig breeding process, which is mainly manifested by the improper use of veterinary drugs. The biggest harm to consumers is the use of banned drugs. In the past years, 32% of pig farm households have used banned drugs. Despite that, the basic conditions for the construction of a pig traceability system in the pig breeding sector are sound and stable, but there are still some problems. A total of 74% of the farm households said they knew the “pork traceability system” or “traceable pork” and 41% considered their pig farms to have participated in the pork traceability system. In total, 73% of pig farm owners stated that all the fattening pigs were wearing ear tags, and 91% of the pig farm owners indicated that they had documented archives for pig breeding or epidemic prevention. A total of 82% of the pig farm households stated that they had obtained animal quarantine certificates when they sold pigs. Furthermore, in addition to the cognitive variables involved in the traceability system, variables such as breeding mode, pig sales mode, and veterinary drug procurement mode also significantly affect the use of veterinary drugs by pig farm households.

The research conclusions in this paper mainly include the following policy implications. First, the government should continue i) to strengthen the construction of a pork traceability system to ensure pork safety and to increase channels to publicize a pork traceability system for pig farm households via training and ii) to improve farm households’ cognition and the level of pork traceability accountability trust, which has a function in regulating the safety behavior of pig farm households. Meanwhile, progress should be made in standardizing and supervising use of ear-tags, use of record-keeping of breeding, and animal quarantine acquisition. The establishment of a pork traceability system provides sound basic conditions. Second, in response to the irregular use of veterinary drugs by pig farm households, the government should also make efforts in encouraging breeding scale and standard management, strengthening supervision and establishing a registration system and a credit rating system for veterinary drug sellers and pig purchasers.

## Figures and Tables

**Table 1:** The definition of independent variables.

Variable Name	Meaning and Valuation	Mean Value	Standard Deviation
Traceability system participation and cognition	Do you think your pig farm has participated in the pork traceability system: Yes = 1, No = 0	0.59	0.49
Use of ear-tags	Are all fattening pigs raised wearing ear tags: Yes = 1, No = 0	0.73	0.44
Use of record-keeping	Whether there are pig breeding archives or epidemic prevention archives in the farm: Yes = 1, No = 0	0.91	0.28
Quarantine certificate acquisition	Whether pigs of the farm obtained the animal quarantine certificate at each sale: Yes = 1, No = 0	0.82	0.39
Working experience	Pig farm households’ working experience (Actual value, unit: Year)	13.52	6.12
Breeding scale	The number of female pigs that can breed at the end of the year: 50 and above = 1, 50 below =0	0.49	0.50
Breeding mode	Whether the farm adopts all-in and all-out breeding mode: Yes = 1, No = 0	0.44	0.50
Slaughtering number	Average number of slaughtering pigs: 50 and above = 1, 50 below = 0	0.47	0.50
Professional cooperatives	Whether to join farm households’ professional cooperatives: Yes = 1, No = 0	0.35	0.48
Pig sales mode	What mode are usually used when selling pigs. Free market trading = 1, agreement or integration = 0	0.56	0.50
Pig sales relationship	Whether there is a fixed and long-term cooperation relationship with pig purchasers in pig acquisition: Yes = 1, No = 0	0.41	0.49
Feed procurement mode	What mode is usually used when purchasing feed. Free market trading = 1, agreement or integration = 0	0.50	0.50
Feed procurement relationship	Whether there is a fixed and long-term cooperative relationship with feed sellers in feed procurement: Yes = 1, No = 0	0.66	0.48
Veterinary drug procurement mode	What mode is usually used to buy veterinary drugs. Free market trading = 1, agreement or integration = 0	0.67	0.47
Veterinary drug procurement relationship	Whether there is a fixed and long-term cooperation relationship with veterinary drug sellers in the purchase of veterinary drugs: Yes = 1, No = 0	0.53	0.50
Understanding level of regulations	How much do you know about feed additives and veterinary drug use regulations: very well, relatively understand = 1, generally understand, poor, very poor = 0	0.67	0.47
Detection level cognition	Do you believe that banned feed additives and veterinary drugs can be detected from pigs: very believe, relatively believe = 1, generally believe, not very believe, distrust = 0	0.89	0.31
Purchaser supervision	How strong is the strength of pig purchasers’ detection and punishment on pork safety, relatively strong = 1, average, weak, very weak = 0	0.65	0.48
Government supervision	How strong is the strength of the government detection and punishment on pork safety: very strong, relatively strong = 1, generally, relatively weak, very weak = 0	0.85	0.36
Gender	Gender: male = 1, female = 0	0.81	0.39
Years	Age (Actual value, unit: Age)	49.49	8.33
Education background	Education background (High school/technical secondary school and above = 1, high school/technical secondary school and below = 0)	0.56	0.50

**Table 2:** Sample distribution.

Provinces and Cities	Urban Area	Sample Number	Proportion	Provinces and Cities	Urban Area	Sample Number	Proportion	Provinces and Cities	Urban Area	Sample Number	Proportion
Henan	Zhumadian	25	6.31%	Hunan	Hengyang	26	6.57%	Beijing	Pinggu	54	13.64%
	Zhengzhou	12	3.03%		Chenzhou	23	5.81%		Shunyi	46	11.62%
	Anyang	11	2.78%		Yongzhou	16	4.04%		Fangshan	40	10.10%
	Luohe	8	2.02%		Shaoyang	11	2.78%		Daxing	26	6.57%
	Nanyang	8	2.02%		Changsha	11	2.78%		Changping	15	3.79%
	Puyang	8	2.02%		Loudi	9	2.27%		Tongzhou	2	0.51%
	Luoyang	7	1.77%		Zhuzhou	7	1.77%				
	Pingdingshan	6	1.52%		Yueyang	6	1.52%				
	Xinyang	6	1.52%		Changde	4	1.01%				
	Jiaozuo	6	1.52%		Huaihua	1	0.25%				
	Kaifeng	1	0.25%		Xiangtan	1	0.25%				

**Table 3:** Basic characteristics of the sample.

Item	Option (s)	Sample Number	Proportion
Gender	Male	322	81.31%
	Female	74	18.69%
Years	18–39 years old	40	10.10%
	40–59 years old	308	77.78%
	60 years old and above	48	12.12%
Education background	Elementary school and below	21	5.30%
	Junior high school	155	39.14%
	High school/technical secondary school	173	43.69%
	Undergraduate/junior college	47	11.87%
	Post graduate	0	0.00%
Working hours	Lower than 5 years	20	5.05%
	5–9 years	74	18.69%
	10–19 years	235	59.34%
	20–29 years	60	15.15%
	30 years and above	7	1.77%
Breeding scale	Lower than 10	51	12.88%
	10–49	144	36.36%
	50–99	80	20.20%
	100 and above	121	30.56%
Breeding mode	All-in and all-out	173	43.69%
	Non-all-in and non-all-out	223	56.31%
Number of pigs sold	Lower than 50	186	46.97%
	50 and above	210	53.03%

**Table 4:** Model estimation results.

Variable Name	Safety Behavior		Traceability System Participation and Cognition	
	Coefficient	Z Value	Coefficient	Z Value
Traceability system participation and cognition	−1.528 ***	−12.84	-	-
Use of ear-tags	-	-	0.324 ***	2.61
Use of record-keeping	-	-	0.338	1.40
Quarantine certificate acquisition	-	-	0.191	1.31
Working experience	0.008	0.73	0.013	1.12
Breeding scale	−0.087	−0.57	0.419 ***	2.57
Breeding mode	−0.212 *	−1.66	−0.034	−0.25
Number of pigs sold	−0.050	−0.34	−0.054	−0.34
Professional cooperatives	−0.029	−0.22	−0.252 *	−1.74
Pig sales mode	0.334 **	2.28	0.046	0.29
Pig sales relationship	−0.020	−0.14	0.135	0.85
Feed procurement mode	−0.072	−0.47	−0.110	−0.67
Feed procurement relationship	0.079	0.52	−0.079	−0.48
Veterinary medicine procurement mode	−0.275 *	−1.68	−0.167	−0.94
Feed procurement relationship	0.148	1.02	−0.025	−0.15
Required level of understanding	0.040	0.29	0.137	0.91
Level of cognition	0.249	1.22	0.249	1.12
Acquirer supervision	0.043	0.32	0.105	0.71
Government supervision	0.218	1.10	0.600 **	2.56
Gender	0.198	1.22	0.361 **	1.97
Age	0.002	0.25	−0.017 *	−1.95
Education background	0.181	1.39	0.201	1.41
Constant term	−0.449	−0.90	−0.675	−1.15
Wald chi^2^	302.37			
Prob > chi^2^	0.0000			

Note: *, ** and *** represent the significance level of 10%, 5%, and 1%, respectively.

**Table 5:** Average marginal effects of the variables in the model.

Variable Name	The Values of Y and T			
	Y = 1, T = 1	Y = 1, T = 0	Y = 0, T = 1	Y = 0, T = 0
Traceability system Participation and cognition	−0.273 ***	−0.172 ***	0.273 ***	0.172 ***
Use of ear-tag	0.035 ***	−0.035 ***	0.072 ***	−0.072 ***
Use of record-keeping	0.037	−0.037	0.075	−0.075
Quarantine certificate acquisition	0.021	−0.021	0.042	−0.042
Working experience	0.003	−0.001	0.001	−0.004
Breeding scale	−0.030	0.055 ***	−0.108	0.083 *
Breeding mode	−0.042	−0.020	0.030	0.031
Number of pigs sold	0.015	−0.0002	0.003	−0.018
Professional cooperatives	−0.033	0.024 *	−0.050 **	0.059
Pig sales mode	0.065 *	0.033 **	−0.050 *	−0.048
Pig sales relationship	0.011	−0.017	0.033	−0.028
Feed procurement mode	−0.025	0.004	−0.011	0.032
Feed procurement relationship	0.006	0.018	−0.032	0.009
Veterinary medicine procurement mode	−0.067	−0.013	0.012	0.068
Feed procurement relationship	0.024	0.019	−0.032	−0.011
Required level of understanding	0.022	−0.010	0.023	−0.035
Level of cognition	0.072	0.001	0.010	−0.083
Acquirer supervision	0.019	−0.007	0.015	−0.028
Government supervision	0.104 *	−0.041	0.094 **	−0.157 **
Gender	0.075 *	−0.017	0.044	−0.102 *
Age	−0.002	0.002 *	−0.004 *	0.004
Education background	0.054	−0.002	0.012	−0.064

Note: *, ** and *** represent the significance level of 10%, 5%, and 1%, respectively.

## Data Availability

The data presented in this study are available on request from the corresponding author. The data are not publicly available as “the rest of the team also needs to write papers with this data”.
